# Is there a link between the neutrophil-to-lymphocyte ratio and venous thromboembolic events after knee arthroplasty? A pilot study

**DOI:** 10.1007/s10195-015-0378-3

**Published:** 2015-09-19

**Authors:** Tyler Barker, Victoria E. Rogers, Vanessa T. Henriksen, Kimberly B. Brown, Roy H. Trawick, Nathan G. Momberger, G. Lynn Rasmussen

**Affiliations:** The Orthopedic Specialty Hospital, 5848 S. Fashion Blvd., Murray, UT 84107 USA; The Orthopedic Specialty Clinic, 5848 S. Fashion Blvd, Murray, UT 84107 USA

**Keywords:** Total knee arthroplasty, Unicompartmental knee arthroplasty, Neutrophil, Lymphocyte, Venous thromboembolic event

## Abstract

**Background:**

This study aimed to identify (1) if the postoperative increase in the neutrophil-to-lymphocyte ratio (NLR) is different between contrasting knee arthroplasty procedures, and (2) if the NLR predicts venous thromboembolism (VTE) after total knee arthroplasty (TKA).

**Materials and methods:**

To address the first objective, we retrospectively studied patients who underwent primary unilateral TKA (*n* = 111) or unicompartmental knee arthroplasty (UKA; *n* = 74) between 2009 and 2012. Patients who required a blood transfusion, underwent autologous blood salvage, experienced any postoperative complication (such as VTE), or were re-admitted >90 days were excluded from analysis. For the second objective, we retrospectively identified patients (cases, *n* = 10) who underwent primary unilateral TKA between 2010 and 2012 and developed postoperative VTE (deep venous thrombosis, pulmonary embolism, or both) during inpatient care (postoperative day 1 or day 2). Cases were matched to surgeon, gender, body mass index, age, and date of surgery controls (*n* = 20) who underwent primary unilateral TKA without developing VTE before patient discharge. The NLR was calculated from the neutrophil and lymphocyte counts extracted from pre- and postoperative (day 1 and day 2) blood chemistry records.

**Results:**

On postoperative day 1, the NLR increase was exacerbated (*p* = 0.02) following TKA compared to UKA and predicted (*p* = 0.02) the occurrence of VTE in TKA patients prior to hospital discharge.

**Conclusion:**

We conclude that the NLR increase is greater following TKA compared to UKA and could serve as a matrix to predict or identify a patient susceptible of sustaining VTE after TKA.

**Level of evidence:**

3.

## Introduction

Osteoarthritis (OA) is the most common degenerative joint disease [1] and the knee is one of the most commonly affected sites. Total knee arthroplasty (TKA) is a safe and effective surgery for treating knee OA, and it is estimated that the number of knee arthroplasty procedures performed annually in the United States will reach 3.5 million by the year 2030 [2]. Although infrequent (~1–3 %), venous thromboembolism (VTE) (including deep venous thromboembolism [DVT], pulmonary embolism [PE], or both) is a life-threatening complication that can occur after TKA [3–6]. Over the past two decades, unicompartmental knee arthroplasty (UKA) has emerged as a less invasive alternative to TKA when knee OA is limited to a single compartment. In comparison to TKA, the prevalence of postoperative VTE (~0.1 %) is apparently lower after UKA [4, 7, 8].

In other pathological conditions, the neutrophil-to-lymphocyte ratio (NLR) is developing as a clinical tool that accurately predicts VTE [9–14]. The NLR is a ratio between the absolute neutrophil and lymphocyte counts, and therefore, is a marker of systemic inflammation representative of innate and adaptive immunity. Although systemic inflammation modulates the coagulation process [15], it is surprisingly unknown if the NLR is different between knee arthroplasty procedures with contrasting risk levels of sustaining VTE, and more importantly, if the NLR predicts VTE after TKA. Therefore, the purpose of this study was two-fold. The first objective was to identify if the NLR is different between arthroplasty procedures that commonly treat knee OA but possess contrasting risk levels of sustaining VTE. The second objective was to identify if the NLR predicts VTE after TKA. We hypothesized that the NLR increase is greater following TKA compared to that after UKA and that the NLR predicts VTE after TKA. We performed two retrospective studies to test our hypothesis. In the first study, we compared the NLR between two knee arthroplasty modalities—total and unicompartmental. The second study consisted of a retrospective case–control design and investigated the predictability of the NLR on VTE after TKA.

## Materials and methods

To test our hypothesis, we performed two studies that were approved by an Institutional Review Board (Central Region, Intermountain Healthcare, Salt Lake City, UT). The studies were performed in accordance with the ethical standards as laid down in the 1964 Declaration of Helsinki and its later amendments or comparable ethical standards.

In Study 1, we retrospectively reviewed the medical charts of 472 patients who underwent elective primary unilateral TKA (*n* = 302) or UKA (*n* = 170) from January 2009 to December 2012 by one orthopedic surgeon at a single institution (The Orthopedic Specialty Hospital, Murray, UT). Patients were identified using the International Classification of Diseases 9th revision, and all patients were diagnosed with knee OA from X-ray images and voluntarily elected for knee arthroplasty. Demographic and surgery variables were recorded for each patient. Patients were excluded from analysis if they underwent bilateral procedures, were re-admitted within 90 days of surgery, had pre- or postoperative infection, had DVT or PE, or received a blood transfusion, manipulation, or autologous blood salvage. Patients were also excluded from analysis if they were lacking an American Society of Anesthesiologists (ASA) score, or the pain and Knee Outcome Survey scores were not documented at physical therapy.

Following screening, 111 TKA (Zimmer Total Knee; Zimmer, Inc., Warsaw, IN, USA) and 74 UKA (Oxford^®^ Partial Knee; Biomet Orthopedics, Warsaw, IN, USA) patients were included in the final analysis. Patients who underwent UKA displayed isolated anteromedial compartment degeneration, a well-preserved lateral compartment, no greater than mild patellofemoral degenerative changes, and an intact anterior cruciate ligament. TKA was performed in patients who did not satisfy the criteria for UKA as determined by one orthopedic surgeon. Tolerable pain levels, normal venous blood O_2_ and blood count, and no signs of PE were required before inpatient discharge after surgery.

In Study 2, we retrospectively identified patients (cases) who underwent elective primary unilateral TKA at a single facility (The Orthopedic Specialty Hospital, Murray, UT) and developed postoperative VTE (i.e., DVT, PE, or both) during inpatient care. Patients who underwent elective primary unilateral TKA without developing VTE during inpatient care served as controls. Cases were identified using the Agency for Healthcare Research and Quality Patient Safety Indicator 12 criteria [16], and the International Classification of Diseases 9th revision. The presence or absence of VTE was confirmed in each patient’s discharge notes. In all cases, DVTs were identified using ultrasound and PEs were identified by chest computed tomography angiogram. Ultrasound and chest computed tomography angiograms were not performed in asymptomatic patients who were identified as controls.

To minimize the variability in surgical procedures, components, and perioperative treatments, data extraction was limited from January 1, 2010 to the end of December 2012. During this period, eleven patients (from 1,339 primary unilateral TKAs; 0.82 %) were identified as experiencing postoperative (day 1 or day 2) VTE. Cases were matched to surgeon, gender, body mass index (BMI), and age controls (two controls for every case). Each case was matched to asymptomatic patients who underwent surgery by the same surgeon and were of the same gender, age, and BMI. Investigators responsible for matching were blinded to blood chemistry results. We were unable to identify controls for one case with an exceptionally high BMI (46.7 kg/m^2^), and therefore, this case was excluded from data analysis. The final analysis consisted of twenty controls and ten cases (DVT, *n* = 5; PE, *n* = 4; DVT and PE, *n* = 1).

Cases were further identified as having a history of diabetes (*n* = 2), heart problems (e.g., previous heart attack, irregular heartbeat, angina, heart failure; *n* = 1), previous blood clot, transfusion or bleeding tendency (*n* = 1), or cancer (*n* = 2). Controls had a history of diabetes (*n* = 4), heart problems (*n* = 10), previous blood clot, transfusion or bleeding tendency (*n* = 5), stroke (*n* = 1), multiple sclerosis (*n* = 1), or cancer (*n* = 2). Controls contained two former and one current smoker, while none of the cases reported a previous or current smoking habit.

The ASA physical status classification score was recorded for each subject. Five subjects (two cases and three controls) received 4–8 mg of dexamethasone before induction of anesthesia. The exclusion of these patients from the statistical analysis did not change the significance of the NLR between groups, and therefore, were included in the final analysis.

All patients were treated with compression stockings from the time of surgery as well as a prophylactic anticoagulant. Ten cases and sixteen controls were titrated with 5 mg of Coumadin^®^ (Bristol-Myers Squibb, New York, NY, USA) in order to achieve an INR target of 1.5–2.0. Two controls were treated with Xarelto^®^ (Leverkusen, Germany) (monitored by anticoagulation monitoring clinic) and two controls with Arixtra^®^ (GlaxoSmithKline, Research Triangle Park, NC, USA) (2.5 mg subcutaneously). Five cases were administered Lovenox^®^ (Sanofi-Aventis, Bridgewater, NJ, USA) when their INR values were <2.0.

For the TKA groups in Study 1 and Study 2, preoperative (morning of surgery and following a 10-h fast) and postoperative (day 1 and day 2; the morning after surgery and following a 10-h fast during inpatient care) blood chemistries were performed at a hospital laboratory (blinded) and results were extracted from an electronic database warehouse. For the UKA group in Study 1, blood chemistries were performed preoperative (morning of surgery and following a 10-h fast) and on day 1 (the morning after surgery and following a 10-h fast during inpatient care). Blood chemistries for the UKA group in Study 1 were limited to postoperative day 1 since a postoperative blood draw on day 2 is not a standard of care procedure for the participating surgeon. The NLR was calculated from the absolute neutrophil and lymphocyte counts.

Data were checked for normality prior to all statistical analyses with the Shapiro–Wilk test. To test the hypothesis for Study 1, and because the data were not normally distributed, we performed a Friedman two-way analysis of variance test followed by multiple pairwise comparisons when appropriate to determine if the NLR was different between TKA and UKA patients prior to and following surgery. Likewise, separate Friedman two-way analysis of variance tests followed by multiple pairwise comparisons when appropriate were performed to determine if the neutrophil and lymphocyte counts were different between TKA and UKA patients prior to and following surgery. To test the hypothesis for Study 2, we performed univariate logistic regression to determine if the NLR on day 1 predicted VTE on day 1 and day 2 post TKA. Separate repeated measures analysis of variance followed by Bonferroni corrections on multiple pairwise comparisons was performed when appropriate on other repeated measure variables for Study 2. The statistical significance of subject characteristics was assessed with separate *t* tests for Study 1 and Study 2. The length of stay and ASA score were assessed with separate Pearson chi-squared tests. All statistical analyses were performed with SYSTAT (version 13.1, Chicago, IL USA). Data are presented as mean (SD) unless noted otherwise.

## Results

**Fig. 1:**
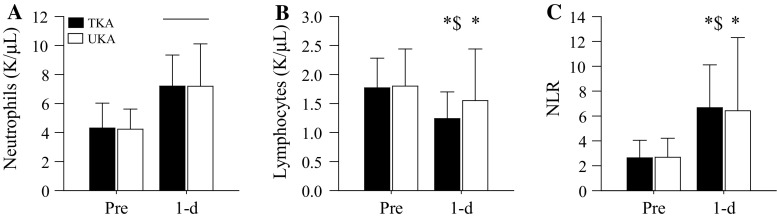
Blood cell counts and NLR prior to (Pre) and one day following (1-d) TKA and UKA. **a** Neutrophil counts were significantly (*bar*, *p* < 0.001 vs Pre) increased following TKA and UKA. The neutrophil increase was not significantly (*p* = 1.00) different between TKA and UKA on 1-d. **b** Lymphocyte counts were significantly decreased following TKA (*p < 0.01 vs Pre) and UKA (*p < 0.01 vs Pre). The lymphocyte decrease was significantly more pronounced (^$^ *p* = 0.001) following TKA compared to that after UKA. **c** The NLRs were significantly increased following TKA (*p < 0.001 vs Pre) and UKA (*p < 0.001 vs Pre). The NLR increase was significantly (^$^ *p* = 0.02) more pronounced after TKA compared to UKA. Data are presented as mean (SD)

Study 1. Blood chemistries and subject characteristics (with the exception of height, *p* = 0.002) were not significantly different (*p* range 0.07–0.90) between TKA and UKA patients prior to surgery (Table 1). As expected, tourniquet duration during surgery was significantly (*p* < 0.001) increased in TKA (69.0 [9.2] min) compared to UKA group [57.8 [5.2] min). Following surgery, neutrophil counts were significantly increased in both groups (*p* < 0.001, Fig. 1a). However, neutrophil counts were not significantly (*p* = 1.00) different between arthroplasty procedures. Conversely, the lymphocyte decrease (*p* = 0.001, Fig. 1b) and NLR increase (*p* = 0.02, Fig. 1c) were more prominent following TKA compared to after UKA.

**Table 1:** Study 1 subject characteristics and results

	TKA	UKA	*P* value
No. (female: male)	111 (80:31)	74 (30:44)	
Age (years)	62 (9)	62 (9)	0.90
Height (cm)	169 (10)	173 (10)	0.002
Body mass (kg)	98.0 (24.7)	95.9 (18.6)	0.80
BMI (kg/m^2^)	34.4 (7.8)	32.1 (5.7)	0.07
ASA score	2.41 (0.58)	2.35 (0.67)	0.34
1 (*n*)	5	7	
2 (*n*)	56	35	
3 (*n*)	50	31	
4 (*n*)	0	1	
Length of stay (days)	2.88 (0.44)	1.22 (0.48)	<0.001
0 (*n*)	0	1	
1 (*n*)	2	57	
2 (*n*)	12	15	
3 (*n*)	94	1	
4 (*n*)	3	0	

Data are presented as mean (SD) unless noted otherwise

**Fig. 2:**
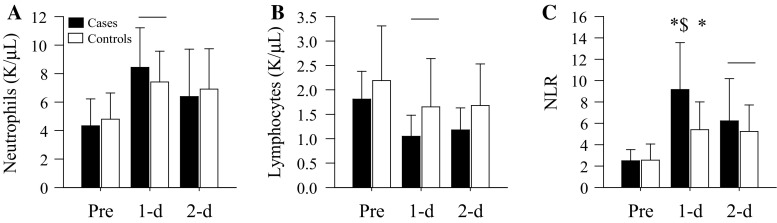
Blood cell counts and the NLR in the cases and controls prior to (Pre) and one and two days following (1-d, 2-d) TKA. **a** Neutrophil counts were significantly increased on 1-d in both groups (*bar*, *p* = 0.008 vs Pre). Neutrophil counts were not significantly different between the cases and control (*p* = 0.22). **b** Lymphocyte counts were significantly decreased on 1-d in both groups (bar, *p* = 0.008 vs Pre). Lymphocyte counts were not significantly different between groups (*p* = 0.32). **c** The NLRs were significantly increased in both groups on 1-d (cases and controls, *p < 0.001 vs Pre) and 2-d (*bar*, *p* ≤ 0.01 vs Pre). The NLR on 1-d was significantly increased (^$^ *p* = 0.002) in the cases compared to the controls. Data are presented as mean (SD)

Study 2. Subject characteristics, length of stay, and blood chemistries prior to surgery were not significantly (*p* range = 0.25–0.78) different between groups (Table 2). On postoperative day 1, there was a significant increase in neutrophil (*p* = 0.008, Fig. 2a) and a significant decrease in lymphocyte (*p* = 0.008, Fig. 2c) counts in the cases and controls. However, neutrophil (*p* = 0.22) and lymphocyte (*p* = 0.32) counts were not significantly different between groups. In contrast, the increase in the NLR was significantly (*p* = 0.002) more pronounced in cases compared to controls on day 1 (Fig. 2c).

**Table 2:** Study 2 subject characteristics and results

	Cases	Controls	*P* value
No. (female male)	10 (7:3)	20 (14:6)	
Age (years)	65 (5)	64 (7)	0.78
Height (cm)	169 (9)	168 (12)	0.74
Body mass (kg)	98.5 (22.2)	91.4 (22.8)	0.43
BMI (kg/m^2^)	34.4 (7.7)	32.4 (7.3)	0.50
ASA score			0.71
1 (*n*)	0	1	
2 (*n*)	6	9	
3 (*n*)	4	10	
Length of stay (days)	3.30 (1.06)	2.90 (0.31)	0.25
2 (*n*)	1	2	
3 (*n*)	7	18	
4 (*n*)	1	0	
6 (*n*)	1	0	
VTE, days from surgery (*n*)			<0.001
1 day	4	0	
2 day	6	0	

Data are presented as mean (SD) unless noted otherwise

Consistent with our hypothesis, the NLR on day 1 predicted the occurrence of VTE after TKA and during inpatient care (*p* = 0.02; odds ratio (OR) 1.38; 95 % confidence interval (CI) 1.05–1.80). We performed a secondary analysis excluding day 1 event cases (*n* = 4) and their controls (*n* = 8) to confirm that a preceding NLR predicts a subsequent VTE after knee arthroplasty. Supporting the earlier finding, the NLR on day 1 predicted VTE on day 2 (*p* = 0.04; OR 1.44; 95 % CI 1.01–2.05).

## Discussion

Knee arthroplasty is an independent risk factor for developing VTE [17–19]. Revealing a matrix from a routine blood chemistry performed prior to and following knee arthroplasty could impact the physician’s decision process, improve patient care, and further minimize the risk of postoperative complications related to VTE without additional costs. In this study, we provide the first evidence that a more invasive knee arthroplasty procedure with an inherently greater risk of sustaining VTE had a more pronounced increase in NLR after surgery. Additionally, the NLR after TKA predicted the subsequent occurrence of VTE prior to hospital discharge. This latter finding suggests that the NLR could serve as an inpatient monitoring tool to assess the risk of VTE. However, considering its uniqueness, future research is needed to confirm or refute the present results and to establish the NLR value that demarcates an increased risk for a VTE.

Previous studies found that an NLR >4.5 predicted coronary heart disease mortality in patients without coronary heart disease [20], while an NLR >4.7 predicted death or myocardial infarction in patients initially assessed for coronary artery disease [14]. In patients screened for or diagnosed with PE, the optimum NLR cut-off value for predicting 30-day mortality was 9.2 [9]. Although the optimum NLR cut-off value provided by Kayrak and colleagues [9] is double the value related to coronary heart disease mortality or myocardial infarction [14, 20], it is similar to the value reported here. However, the NLR that predicted VTE after TKA is an acutely elevated value while the values previously reported in other patient populations (i.e., non-orthopedic) are presumably chronic. Clearly, additional research delineating the condition-specific NLR that predicts clinical outcomes and the prognostic role of acute NLR fluctuations on VTE is warranted.

Another unique finding of the present investigation was the exaggerated increase in the NLR following TKA compared to UKA. The exaggerated increase in the NLR was mediated by the dramatic and more severe presence of lymphopenia following TKA. An increase in the NLR and lymphopenia are predictive of cardiovascular events, such as acute myocardial infarction (AMI), VTE, and overall mortality in cardiac patients [10–14]. Despite the use of thromboprophylaxis, the risk of AMI (~0.4 %), VTE (~1.1 %), and major bleeding (~0.5 %) after TKA substantially increase within 90 days of surgery [3, 5, 21]. Extrapolating from TKA data, evidence suggests that UKA results in fewer complications in terms of DVT [4, 7, 8]. Although the percentage of TKA and UKA patients affected by such devastating cadiovascular ailments is low, these are impactful consequences and further research investigating the prognostic role of the NLR on such life-changing events after knee arthroplasty is warranted.

Although the present study provides novel results, there are a few limitations worthy of discussion. First, in Study 2, computed tomography and ultrasound were not performed in the asymptomatic control group. Therefore, it is unknown if the control group had silent DVT or PE. In line with this limitation, data extraction and analysis were limited to perioperative outcomes and it is unknown if any of the controls developed VTE following discharge. Second, Study 1 consisted of a small sample size; however, this was expected when considering the infrequency of a VTE. Third, in Study 2, 39 % of the subjects underwent UKA and the delineation of performing UKA was determined by a single orthopedic surgeon with a predefined standard of care screening protocol. This approach strengthens the interpretation between patients with contrasting knee abnormalities, but it constrains the broad application of these findings to other studies conducted in patients who underwent TKA who could have been candidates for UKA. Finally, although the NLR difference between UKA and TKA groups is likely the result of the surgical procedures, it would be remiss to ignore the underlying pathological differences between groups prior to arthroplasty. Future research is also needed to identify if the type of prosthesis, as well as different types of anticoagulant medications, impact the assessment of the NLR on VTE following knee arthroplasty.

Despite low rates (~1–3 %), the absolute number of patients who will sustain VTEs is anticipated to increase with the expected continuous growth in the number of TKA procedures performed annually. Thus, developing a matrix that improves prognostic potential could alter patient treatment strategies and further minimize morbidity and mortality rates following knee arthroplasty. In this study, the NLR increase was exacerbated following a more invasive knee arthroplasty procedure (i.e., TKA) characterized by an increased risk of VTE. Moreover, the NLR following TKA predicted VTE prior to patient discharge. Based on these findings, we conclude that NLR increase could serve as a matrix for monitoring the susceptibility of developing VTEs after knee arthroplasty and prior to hospital discharge. Confirmation of the preliminary data reported here is essential and could be paramount in guiding the physician’s treatment methods during inpatient care following knee arthroplasty.
